# Hepatitis B reactivation characterized by HBsAg negativity and anti-HbsAg antibodies persistence in haematopoietic stem cell transplanted patient after lamivudine withdrawal

**DOI:** 10.1186/s12879-017-2672-6

**Published:** 2017-08-15

**Authors:** C. Cerva, G. Maffongelli, V. Svicher, R. Salpini, L. Colagrossi, A. Battisti, B. Mariotti, R. Cerretti, L. Cudillo, L. Sarmati

**Affiliations:** 1 Clinical Infectious Disease, Department of Systems Medicine, Tor Vergata University, Rome, Italy; 2 Department of Experimental Medicine and Surgery, Tor Vergata University, Rome, Italy; 3 Department of Hematology, Stem Cell Transplant Unit, Tor Vergata University, Rome, Italy

**Keywords:** HBV reactivation, Hbv DNA, Prophylaxis, Hepatitis B, Hematopoietic stem cell transplantation

## Abstract

**Background:**

HBV reactivation is associated with high mortality rates in hematopoietic stem cell transplantation (HSCT) and prophylactic lamivudine (LMV) treatment is suggested to prevent this phenomenon. However, the duration of LMV treatment in HSCT patients is not fully defined and the time of immune recovery is considered the best parameter for a drug to be safely interrupted. In patients undergoing allogeneic HSCT, the time of immune recovery is not easy to define and may take years after transplantation and prolonged LMV treatments, which can lead to drug-resistant viral strains.

**Case presentation:**

An anti-HBc-positive hematological patient who was undergoing prolonged immunosuppression and who experienced HBV reactivation 3 months after the suspension of a prolonged LMV prophylaxis is described. HBV-DNA matching an atypical serological profile characterized by HbsAg negativity and anti-HBs positivity was detected in the patient. The genotypic analysis of the HBV strain identified T127P, F170FL and S204R mutations of HbsAg, which can hinder HBsAg recognition in a diagnostic assay.

**Conclusions:**

HBV reactivation in the HSCT host can be sustained by HBsAg viral variants with characteristics of altered immunogenicity that cannot be detected by usual laboratory tests. This clinical case description suggests the importance of screening for serum HBV-DNA levels in the diagnosis of HBV reactivation and monitoring HBV-DNA after prophylaxis suspension, particularly in HSCT subjects who have undergone prolonged periods of LMV treatment.

## Background

HBV reactivation is a complication of immunosuppressive treatments and is associated with high mortality [1]. Patients undergoing allogeneic hematopoietic stem cell transplantation (allo-HSCT) are considered at major risk for HBV reactivation [1–6], with a mortality rate of up to 40% [3]. A number of national and international guidelines [7–9] have addressed the prophylaxis of HBV reactivation in an immunocompromised host. Nonetheless, several aspects of HBV prevention, such as prophylaxis duration and virological monitoring after prophylaxis suspension, remain poorly defined, particularly in HSCT cases, where HBV reactivation may occur several years after the start of immunosuppressive treatments [10]. Prolonged lamivudine (LMV) prophylaxis is associated with the occurrence of LMV resistance at a rate that increases to 60% in immunocompromised patients [11]. Viral variants, both resistant and sensitive to LMV, can develop in patients under anti-viral treatment and may lead to HBsAg amino acid changes with altered antigenicity, also known as immune escape mutants. The modified HBsAg produced by these viruses is not detectable by current commercial assays.

Here, we report the case of a severely immunocompromised allo-HSCT patient who experienced reactivation of HBV infection a few months after the withdrawal of a very long-term LMV prophylaxis, without the reappearance of HBsAg and with the persistence of high titer anti-HBs antibodies.

## Case presentation

The patient is a 59-year-old Italian male who received a diagnosis of non-Hodgkin’s lymphoma (NHL) (stage IV) in May 2003. Histological diagnosis indicated a small B-cell lymphoma. From September 2003 to January 2004, he underwent 6 R-CHOP courses (rituximab, cyclophosphamide, doxorubicin, vincristine and prednisone), which were followed by complete disease remission. In March 2005, the patient experienced an NHL relapse. Therefore, fludarabine treatment (4 courses) was started, and the treatment again resulted in complete remission. In November 2005, he underwent an autologous HSCT. In May 2010, a second relapse of NHL was diagnosed, and a treatment regimen with Rituximab-Bendamustine (R-Bendamustine) was started. After 6 courses of R-Bendamustine, a partial response was obtained. The disease had progressed again by October 2011, and an R-DHAP (rituximab, cisplatin, cytosine arabinoside and dexamethasone) treatment was started. Because of renal toxicity, the R-DHAP was replaced with Alentuzumab, obtaining a partial response. In February 2012, the disease had progressed again. Ofatumumab treatment (9 courses) was initiated with no clinical response. In November 2012, the patient underwent an allo-HSCT by a matched unrelated donor (MUD) as a curative option due to refractory disease. The patient received a reduced intensity condition regimen ([TBF-RIC] consisting of thiotepa, busulfan, and fludarabine plus anti-thymocyte globulin). Graft versus host disease (GvHD) prophylaxis consisted of cyclosporine and methotrexate. The engraftment for neutrophils >500/mmc and platelets >20,000/mmc occurred 20 and 24 days after the allo-HSCT, respectively. The post-transplant course was complicated by Aspergillus pneumonia (day 19 post allo-HSCT), for which the patient underwent prolonged antifungal treatment. In July 2013, the patient had the residual pulmonary nodule surgically removed. In the context of the fungal nodule, a lung adenocarcinoma was histologically present.

After allo-HSCT, the patient achieved a stable, complete remission of hematological disease (last follow-up visit in May 2016). At the HBV screening, the patient was identified as positive for anti-HBs, anti-HBc and anti-HBe, and negative for HBV-DNA. The HBV donor serology showed no previous HBV infection (anti-HBc/anti-HBs negative). An HBV DNA test was performed that resulted in a negative outcome. He was not vaccinated for HBV.

Therefore, LMV prophylaxis was started in 2010. The patient continuously received LMV for up to 24 months after the allo-HSCT (January 2015), at which point the prophylaxis was suspended. During the LMV prophylaxis, he remained positive for anti-HBs, anti-HBc and anti-HBe, and HBV-DNA was consistently undetectable. Three months after the suspension of LMV treatment (March 2015), he experienced virological HBV reactivation (serum HBV DNA 42 IU/mL), and no other HBV serological variations were present. Despite HBV reactivation, HBsAg remained negative, and the anti-HBs titers remained consistently high (482 mIU/ml in November 2014, 505 mIU/ml in March 2015, 457 mIU/ml in April 2015, 576 mIU/ml in June 2015 and 542 mIU/ml in July 2015).

A genotypic test (based on the sequencing of HBV reverse transcriptase and HBsAg) was performed during the HBV reactivation diagnosis. The test did not reveal the presence of LMV resistance mutations but showed the presence of three mutations in HbsAg: T127P, F170FL and S204R.

**Fig. 1:**
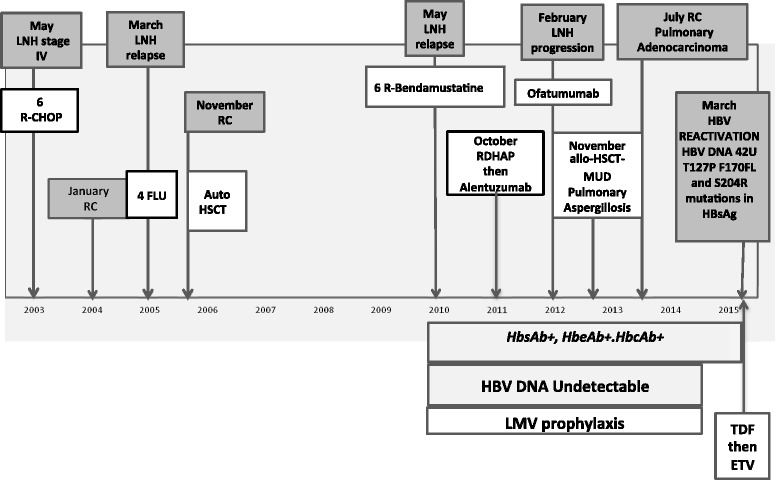
Medical History. LNH: non-Hodgkin’s lymphoma; R-CHOP: rituximab, cyclophosphamide, doxorubicin, vincristine and prednisone; FLU: fludarabine treatment; RC: complete remission of disease; Auto HSCT: autologous peripheral blood stem cell transplantation; R: rituximab; RDHAP: rituximab, cisplatin, cytosine arabinoside and dexamethasone; Allo-HSCT: allogenic peripheral blood stem cell transplantation; MUD: matched unrelated donor

Due to the persistence of quantitative HBV DNA (35 IU/mL), antiviral therapy was started with 245 mg tenofovir. After 2 weeks, the patient underwent a bone density test that documented osteopenia, while the glomerular filtration rate remained stable, although reduced. Tenofovir treatment was interrupted and 0.5 mg entecavir was started. The patient is now in complete hematological remission, and the quantitative HBV DNA is undetectable (see Fig. 1).

## Conclusions

This paper describes the clinical case of an anti-HBc-positive and anti-HBs-positive patient undergoing prolonged immunosuppression who developed HBV reactivation 3 months after the suspension of prolonged (4 years) LMV prophylaxis. At HBV reactivation, the patient showed an atypical serological profile characterized by HBsAg negativity and anti-HBs positivity (with a high antibody titer of 505 mIU/ml). These results corroborate recently published studies showing that a substantial proportion (10% to 80%) of patients who tested positive for anti-HBc and anti-HBs remained HBsAg-negative despite the reuptake of viral replication [12–14]. Overall, our results suggest that this immunological profile is critical in the management of patients who are at risk of HBV reactivation and strongly support the use of serum HBV-DNA (rather than HBsAg) for the diagnosis of HBV reactivation.

HBsAg negativity may be related to the high degree of genetic variability in HBsAg observed in patients who develop immunosuppression-driven HBV reactivation, which may hinder HBsAg recognition by the antibodies used in the diagnostic assay [12, 13]. In particular, a previous study has shown the enrichment of additional N-linked glycosylation sites in the major hydrophilic HBsAg region in patients who remained HbsAg-negative despite HBV reactivation [10]. In vitro studies have shown the ability of these glycosylation sites to hamper or abrogate the recognition and quantification of HBsAg by the currently available diagnostic test. In the current clinical case, the HBsAg mutation S204R was detected. This mutation was localized in the C-terminal transmembrane domain, which is known to play an important role in the modulation of HBsAg secretion and viral particle assembly [15]. The acquisition of a positively-charged amino acid (Arginine [R] at position 204) in this transmembrane domain might alter the HBsAg structure to result in i) HBsAg negativity in the currently available diagnostic test and ii) HBV escape from a high anti-HBs titer. This mutation may also affect the viral particle release, resulting in HBV reactivation that is characterized by a low level of serum HBV DNA, as demonstrated in our clinical case. This concept agrees with a previous study, which showed that the viral clones encoding S204R (lysine [S] substitution with arginine [R] at position 204) and G145R (glycine [G] substitution with arginine [R] at position 145) exhibited an approximately 60% viral secretion defect compared with the wild-type. According to this result, the authors proposed that the basic arginine residues may be important for HBsAg retention within the endoplasmic reticulum [16].

Finally, the genotypic testing also highlighted the presence of two HBV RT mutations (S135Y and I233V) that are potentially involved in resistance to first-generation nucleos(t)ide RT inhibitors [17, 18]. The role of these genetic mutations in facilitating HBV evasion from LMV warrants further investigation.

This clinical case also demonstrates the challenges of determining the proper duration of prophylaxis. The current guidelines recommend suspending prophylaxis from the immunosuppressive treatments after 12–18 months [19]. However, in the setting of HSCT, the risk of HBV reactivation can persist for several years after transplantation due to the long delays in immune reconstitution. In this context, Hammond et al. [20] showed that the cumulative probability of HBV reactivation increases from 9%, 1 year after transplantation, to 43%, 4 years after transplantation. Thus, the duration of prophylaxis needs to be extended further in the setting of profound immunosuppression to prevent late HBV reactivation episodes. In addition, these findings also highlight the need for markers that can predict the full immune reconstitution against HBV.

In conclusion, the described case demonstrates that HBV reactivation in the immunocompromised host is a complex phenomenon often sustained by virus strains with virological characteristics that can prevent the recognition of the virus by specific antibodies and by the usual laboratory tests. Therefore, we recommend the careful consideration of the duration of HBV prophylaxis and the subsequent virological monitoring, which should also include the measurement of viral DNA in these patients.

## Abbreviations

HSCT: Hematopoietic stem cell transplantation
MUD: Matched unrelated donor
NHL: Non-Hodgkin’s lymphoma
PBSCT: Peripheral blood stem cell transplantation
R: Rituximab
R-CHOP: Rituximab, cyclophosphamide, doxorubicin, vincristine and prednisone
RDHAP: Rituximab, cisplatin, cytosine arabinoside and dexamethasone
TBF-RIC: Thiotepa, busulfan, and fludarabine reduced intensity condition regimen
